# Inter and intra-hemispheric structural imaging markers predict depression relapse after electroconvulsive therapy: a multisite study

**DOI:** 10.1038/s41398-017-0020-7

**Published:** 2017-12-08

**Authors:** Benjamin S. C. Wade, Jing Sui, Gerhard Hellemann, Amber M. Leaver, Randall T. Espinoza, Roger P. Woods, Christopher C. Abbott, Shantanu H. Joshi, Katherine L. Narr

**Affiliations:** 1 Department of Neurology, UCLA, Ahmanson-Lovelace Brain Mapping Center, Los Angeles, USA; 2 Department of Psychiatry and Biobehavioral Sciences, UCLA, Los Angeles, USA; 3 The Mind Research Network and Lovelace Biomedical and Environmental Research Institute, Albuquerque, NM USA; 4 Brainnetome Center and National Laboratory of Pattern Recognition, Institute of Automation, Chinese Academy of Sciences, Beijing, China; 5 Chinese Academy of Sciences Center for Excellence in Brain Science, Institute of Automation, Beijing, China; 6 Department of Psychiatry and Biobehavioral Sciences, Semel Institute, UCLA, Los Angeles, USA; 7 Department of Psychiatry, University of New Mexico, Albuquerque, USA

## Abstract

Relapse of depression following treatment is high. Biomarkers predictive of an individual’s relapse risk could provide earlier opportunities for prevention. Since electroconvulsive therapy (ECT) elicits robust and rapidly acting antidepressant effects, but has a >50% relapse rate, ECT presents a valuable model for determining predictors of relapse-risk. Although previous studies have associated ECT-induced changes in brain morphometry with clinical response, longer-term outcomes have not been addressed. Using structural imaging data from 42 ECT-responsive patients obtained prior to and directly following an ECT treatment index series at two independent sites (UCLA: *n* = 17, age = 45.41±12.34 years; UNM: *n* = 25; age = 65.00±8.44), here we test relapse prediction within 6-months post-ECT. Random forests were used to predict subsequent relapse using singular and ratios of intra and inter-hemispheric structural imaging measures and clinical variables from pre-, post-, and pre-to-post ECT. Relapse risk was determined as a function of feature variation. Relapse was well-predicted both within site and when cohorts were pooled where top-performing models yielded balanced accuracies of 71–78%. Top predictors included cingulate isthmus asymmetry, pallidal asymmetry, the ratio of the paracentral to precentral cortical thickness and the ratio of lateral occipital to pericalcarine cortical thickness. Pooling cohorts and predicting relapse from post-treatment measures provided the best classification performances. However, classifiers trained on each age-disparate cohort were less informative for prediction in the held-out cohort. Post-treatment structural neuroimaging measures and the ratios of connected regions commonly implicated in depression pathophysiology are informative of relapse risk. Structural imaging measures may have utility for devising more personalized preventative medicine approaches.

## Introduction

Major depressive disorder (MDD) has a lifetime prevalence of 16% in the U.S., with comparable high rates in other nations^1^. Approximately 40% of depressed patients do not respond to standard first-line treatments^2,3^. Following the first major depressive episode, >50% of recovering patients suffer relapse and >15% experience unremitting, chronic symptoms^4^; recurrence will occur in ~80% of patients with a history of two or more prior episodes^5–7^.

The high relapse and recurrence rates, usually defined as deterioration to the full disease syndrome during a period of remission or the appearance of a new episode after a longer period of recovery, respectively^8^, underscore the need for timely intervention. Identifying clinical, demographic, and physiological markers predictive of relapse and symptom recurrence are thus critical for prevention. Previous reports have associated incomplete recovery^9^, illness duration and number of previous episodes with long-term clinical outcomes^8,10^. Fewer studies have related neuroimaging measures to clinical outcome following treatment. Of these, the majority have focused on prediction of acute response or remission directly following treatment. Even less have attempted to predict a patient’s likelihood of relapse/recurrence in the months following treatment.

Of prior studies addressing neuroimaging markers of long-term clinical outcomes following antidepressant treatment, one investigation following 30 MDD patients prospectively over 3-years reported that those with smaller hippocampal volumes and recurrent depression had worse outcomes, irrespective of continued medication status^11^. In a follow-up study, investigators showed that patients who remitted during the 3-year timeframe had less atrophy of the left hippocampus, left anterior cingulate (ACC), and left dorsomedial prefrontal cortex, lateralized to the left hemisphere, and bilateral dorsolateral prefrontal cortex, than non-remitters^12^. Corroborating evidence from a recent study following 49 MDD patients over 5-years similarly found patients with smaller ACC volumes (though right-lateralized) prior to treatment had poorer clinical outcomes^13^. Further, inclusion of volumetric measures of both the right ACC and right inferior frontal cortex increased the explained variance for change in mood by 20% over inclusion of clinical and demographic measures alone. These findings support the potential utility of structural imaging measures as biomarkers of clinical outcome^13^.

Using naturalistic designs, others have shown that task-related brain activation within dorsolateral and medial prefrontal regions, striatum, and parietal regions are predictive of a chronic versus a more favorable clinical course of depression with up to 73% accuracy^14^. Interestingly, structural imaging measures were not predictive in this particular study. Another group also found that activation of the ACC and ventromedial prefrontal cortex during an emotional challenge task was strongly associated with increased relapse risk ~18-months following remission. Using a signal change threshold of ≥ 0% and regions-of-interest targeted from statistical remitter-control contrasts, relapsing patients were classified with a sensitivity and specificity of 90% and 83%, respectively^15^.

Notably, while these studies made important strides towards identifying biomarkers of clinical outcome, with few exceptions^14^, these investigations tested for post-hoc associations rather than the predictive utility of imaging biomarkers in a cross-validation framework^11–13,15^. Further, prospective studies have followed subjects within a naturalistic setting where patients have received several possible treatment regimens^13,14^. Generalization across treatments is important, but since different treatment strategies may have different response/relapse trajectories, different degrees of compliance and differentially affect a patient’s neurobiology, focus on a single treatment modality may be beneficial for targeting neural predictors of relapse and symptom recurrence in MDD.

Here, we focus on predicting relapse/recurrence within 6-months following treatment with electroconvulsive therapy (ECT), which is a well-established treatment typically reserved for patients with severe treatment-resistant depression. ECT works more quickly (response can occur in 2–4 weeks) and has higher remission rates than other standard therapeutic approaches^16^. However, relapse/recurrence rates of depression after ECT are similar to those of other antidepressant treatments. Following ECT, relapse occurs in ~50% of patients with most relapsing within the first 6-months^17^. The fast acting and robust clinical effects of ECT together with relapse and recurrence risks comparable to other treatments make this treatment ideal for determining whether variations in brain morphometry are predictive of individual clinical outcome.

Selecting 42 patients who initially responded to ECT from two studies conducted at independent sites, the University of California, Los Angeles (UCLA) and the University of New Mexico (UNM), we developed a random forest (RF) classifier to identify structural neuroimaging, clinical, and demographic factors predictive of symptom recurrence/relapse. Imaging measures were derived from pre-treatment, post-treatment, and pre-post treatment change in subcortical volumes and regional cortical thickness and ratios between these measures. The motivation for the latter included the following: (i) ratios for cortical thickness/volumes are normalized within subject, (ii) they capture effects of asymmetry since the ratios of homologous regions are included, and (iii) they represent structural relationships between spatially diffuse regions in a spirit similar to modeling structural networks. Because ECT was administered unilaterally in the majority of patients, we hypothesized that measures of asymmetry would relate to relapse risk.

The identification of a set of biomarkers informing a patient’s probability of symptom recurrence following treatment is of high translational value. Clinicians could refine maintenance strategies for at-risk individuals to prevent relapse, which could offset the disproportionately high cost of managing recurrent major depression.

## Patients and Methods

### Participants

Patients experiencing a DSM-IV defined major depressive episode and eligible to receive ECT were recruited from UCLA (*N* = 42) and UNM (*N* = 40). All patients received structural MRI scans and mood evaluations 24 h prior to ECT (pretreatment) and within a week of completing ECT index (post-treatment). Patient mood was again assessed approximately 6-months following index (follow-up). The Hamilton Depression Rating Scale (HAM-D-17)^18^ tracked symptomology at each time point. A patient was defined to have relapsed if (i) their HAM-D reduced by ≥ 50% over ECT index indicating therapeutic response and (ii) their long-term follow-up HAM-D score was ≥ 17. The threshold for determining response was selected since it the most commonly used definition of clinically meaningful response^19^. Definitions of relapse following initial response are less consistent. Since the current study (a) included patients with severe and treatment resistant depression, (b) the criterion used for response still allowed for some residual symptoms, and (c) HAM-D scores of ≥ 17 are considered a cut-off for separating mild^8–16^ from moderate^17–23^ and severe depression ( ≥ 24)^20^, a HAM-D score of ≥ 17 at final follow-up was used to define relapse. A total of 17 patients at UCLA (10 females, mean age = 45.41 ± 12.34) and 25 at UNM (18 females, mean age = 65 ± 8.44) were defined as ECT responders and included in the study. Following the ECT index, patients continued on naturalistic course of maintenance therapy. Ten UCLA patients and 11 at UNM received maintenance or continuation (m-/c-ECT), respectively. Within 6 months following ECT index 6 patients (35%) relapsed at UCLA while 13 (52%) relapsed at UNM.

All patients had experienced two or more earlier major depressive episodes and failed to respond to at least two prior adequate medication trials in the index episode. Exclusionary criteria included first-episode depression, diagnosed neurological or neurodegenerative disorder, any head injury with loss of consciousness over 5 min, comorbid psychiatric conditions such as schizophrenia or schizoaffective disorder, current drug or alcohol abuse (excluding nicotine), and MRI contraindications. Bipolar disorder was exclusionary at UNM but not at UCLA, though mania in the index episode was exclusionary at UCLA. UCLA patients were excluded if the age of depression onset was over 50 years. UCLA patients were tapered off of antidepressants and benzodiazepines in preparation for ECT and were completely free of medication for at least 48–72 h before enrollment and ECT treatment. The UNM cohort was not tapered off of medication before ECT. All participants provided written informed consent as approved by the UCLA or UNM Institutional Review Board.

### MR acquisition

At UCLA, high-resolution motion-corrected multi-echo T1-weighted MPRAGE structural brain images^21^ were acquired on a Siemens 3 T Allegra system (Erlangen, Germany) for all subjects and time points (TEs/TR = 1.74, 3.6, 5.46, 7.32/2530 ms, TI = 1260 ms, flip angle = 7°, voxel resolution = 1.3 × 1 × 1 mm^3^). Patients at UNM were scanned on a 3-Tesla Siemens Trio scanner with a similar multi-echo T1-weighted MPRAGE (TR = 2.53 s (s), TE = 1.64, 3.5, 5.32, 7.22, 9.08 ms, TI = 1.20 s, flip angle = 7°, number of excitations = 1, and voxel resolution = 1 × 1 × 1 mm^3^).

### Image preprocessing

Validated FreeSurfer^22^ workflows, including removal of non-brain tissue, intensity normalization and automated volumetric parcellation based on probabilistic information from manually labeled training sets, were used for whole brain cortical (Desikan Killiany atlas-based parcellations^23^) and subcortical segmentation. Each segmentation was visually inspected to ensure its quality.

### Candidate features

Clinical, demographic and structural neuroimaging features of each participant were included as candidate features for the RF classifier. Demographic information included age and sex; clinical information was comprised of pre- and post-treatment HAM-D-17 scores, number of ECT sessions received, and electrode placement (right unilateral or bilateral). Maintenance therapy status (m-/c-ECT) was not a predictor. Imaging features included volumes of the accumbens, amygdala, caudate, hippocampus, pallidum, putamen, thalamus, and lateral ventricles and the mean thickness of 34 homologous cortical regions.

In addition to individual measures of subcortical volumes and cortical thickness we included pairwise ratios of each subcortical volume to every other subcortical volume and likewise for cortical thickness. These ratios have several important benefits. First, they are normalized within subject and thus robust to confounds of age- and sex-related group differences. Ratio measures additionally capture the asymmetry of homologous regions that may reflect underlying neurobiological properties related to clinical outcome. Second, the distribution of ECT electrode placements largely determines the electrical field distribution within the brain and the distribution of ECT’s direct effects. Since unilateral electrode placement is mostly applied at UCLA and UNM, there are likely to be highly lateralized effects, which may also relate to clinical outcome.

### Predictive modeling

We used supervised RFs^24^ to classify relapse status based on either pre- or post-treatment features or changes in these features over treatment. RFs were used due to their robustness against overfitting in high-dimensions, minimal tuning parameters, and overall competitive performance relative to other state-of-the-art methods^25^. Alternative classifiers were not compared to RFs in order to mitigate type 1 errors. We considered models built from participants exclusively at UCLA or UNM and observations combined across sites. Models fit to a single cohort were additionally assessed by predicting relapse across site.

We implemented leave-one-out cross-validation (LOO-CV) to validate our classifier. Within each iteration of LOO-CV, the set of N-1 participants used to train the model was partitioned into 10 nested folds to further randomize the feature selection process which consisted of multiple steps. We first held out one of the 10 folds. Second, features in the remaining folds were subjected to a collinearity filter in which we identified pairs of features correlated above a threshold, |*r*|. The feature in this pair with the highest correlation with the remaining features in the dataset was excluded. The optimal threshold, |*r*|, was found using a grid search over the space r ∈ {0.1, 0.2,…, 1.0}. The remaining features were then passed to a recursive feature elimination^26^ (RFE) algorithm where the internal classifier was a RF composed of 1000 trees and the feature set maximizing classification specificity was selected.

**Fig. 1:**
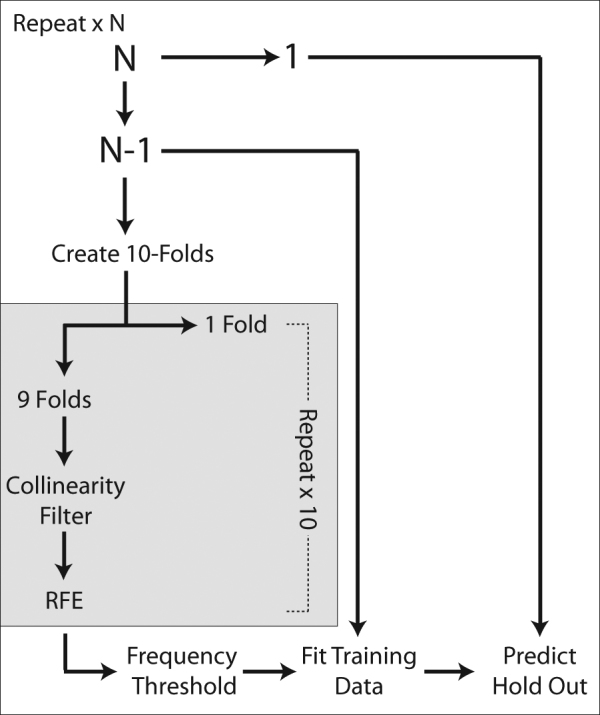
Flowchart of classification process At each leave-one-out cross-validation fold, training subjects are randomly assigned to each of ten folds and feature selection based on collinearity filtering and recursive feature elimination (RFE) is performed on nine of the folds. Features selected by this process above a given threshold are used to train a model on the whole training set and the parameters from this model are used to predict the originally held-out observation

After this process was repeated 10 times, we evaluated the frequency with which each feature was selected by RFE. We retained features selected above the ⍴-th quantile of frequencies where ⍴ ∈ {0.1, 0.2,…, 1.0}. Finally, the features identified by this process were used to fit a RF to the entire N-1 set of training observations. RF-specific tuning parameters are outlined in Supplementary Methods. The final RF predicted the originally held-out observation (Fig. 1). A grid search was performed over the space of parameters *r* and ⍴ for each location and time-point specific model. Models constructed within-site were additionally used to predict the patient relapse across site as further validation. All RFs were implemented using the caret^27^ package in R version 3.3.2^28^.

Performance measures in binary classification with imbalanced class proportions are biased towards the more prevalent class. To avoid reporting optimistic performances we report the balanced accuracy (BA)^29^ defined as the arithmetic mean of sensitivity and specificity. We additionally compare the BA to the baseline detection rate (BDR), which is the accuracy obtained by hypothetically assigning each unknown patient to the most prevalent class. The BDR is naturally >50% in problems with imbalanced classes.

Two post-hoc analyses were also conducted (see Supplementary Methods and Results). First we explored our model’s performance as a function of classifying only patients for which the classifier had more than a particular level of confidence, known as classification with a rejection option^30^. We secondly repeated our entire analysis using 10 repeated 10-fold cross-validation to assess the classifier’s robustness across cross-validation schemes.

### Code availability

Code developed for these analyses are available upon request.

## Results

### Demographic and clinical measures

Table 1 shows patient demographic and clinical characteristics. The UNM cohort was significantly older than the UCLA cohort. Post-treatment HAM-D scores were significantly lower at UNM. However, the cohorts did not differ significantly by sex, average number of ECT sessions, proportion of patients receiving RUL electrode placement, pretreatment HAM-D or the proportion relapsers.

**Table 1:** Demographic and clinical characteristics

	UCLA, *N* = 17	UNM, *N* = 25	*t* or *χ* ^2^ (*p*-value)
Age, mean (SD), years	45.41 (12.34)	65.00 (8.44)	*p* = 5.34e-06
Sex, (M/F)	7/10	7/18	*p* = 0.57
# of ECT index sessions, mean (SD)	10.11 (2.31)	10.36 (3.05)	*p* = 0.77
# of RUL arrangements, (RUL/BT)	16/1	21/4	*p* = 0.61
Pretreatment HAM-D-17, mean (SD)	27.29 (5.24)	26.44 (7.26)	*p* = 0.66
Post-treatment HAM-D-17, mean (SD)	6.82 (3.28)	3.12 (3.07)	*p* = 0.00082
# Relapsers/Non-relapsers	6/11	13/12	*p* = 0.45
# Unipolar/Bipolar	14/3	25/0	*p* = 0.11
	UCLA Relapsers, *N* = 6	UCLA Non-relapsers *N* = 11	Significance
Age, mean (SD), years	43.16 (13.89)	46.63 (11.94)	*p* = 0.61
Sex, (M/F)	0/6	7/4	*p* = 0.04
# of ECT index sessions, mean (SD)	10.00 (1.67)	10.18 (2.67)	*p* = 0.86
# of RUL arrangements, (RUL/BT)	6/0	10/1	*p* = 1
Pretreatment HAM-D-17, mean (SD)	28.83 (4.79)	26.45 (5.50)	*p* = 0.37
Post-treatment HAM-D-17, mean (SD)	6.66 (2.65)	6.90 (3.70)	*p* = 0.87
# Maintenance ECT, (Yes/No)	5/1	5/6	*p* = 0.30
	UNM Relapsers, *N* = 13	UNM Non-relapsers *N* = 12	Significance
Age, mean (SD), years	65.15 (9.76)	64.83 (7.17)	*p* = 0.92
Sex, (M/F)	3/10	4/8	*p* = 0.90
# of ECT index sessions, mean (SD)	10.53 (3.38)	10.16 (2.79)	*p* = 0.76
# of RUL arrangements, (RUL/BT)	10/3	11/1	*p* = 0.64
Pretreatment HAM-D-17, mean (SD)	25.92 (6.10)	27.00 (8.60)	*p* = 0.72
Post-treatment HAM-D-17, mean (SD)	3.23 (3.46)	3.00 (2.73)	*p* = 0.85
# Continuation ECT, (Yes/No)	6/7	5/7	*p* = 1

UCLA University of California, Los Angeles, UNM University of New Mexico, ECT Electroconvulsive therapy, RUL Right unilateral electrode placement, BT Bi-temporal, HAM-D Hamilton depression rating scale.

All relapsing patients were female at UCLA; 10 of the 13 relapsing patients were female at UNM. This proportion did not differ significantly from non-relapsers. Across both sites, relapsers did not differ significantly from non-relapsers in terms of pre- or post-treatment HAM-D scores, number of ECT sessions received, proportion of patients receiving right unilateral electrode placement, age, or m-/c-ECT status.

### Prediction from pre-treatment measures

**Fig. 2:**
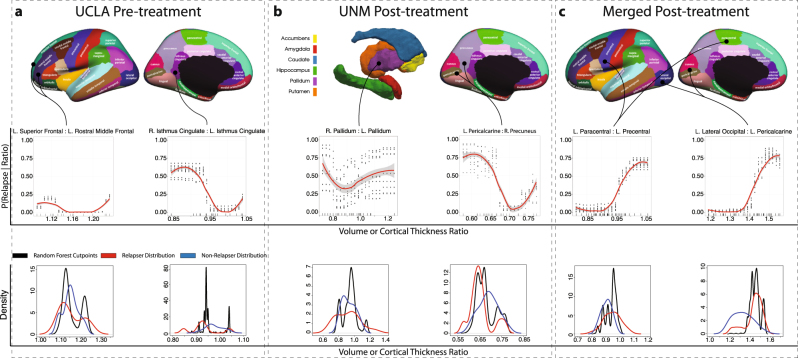
Regions predictive of relapse and their relationship to the predicted probability of relapse Top row illustrates anatomical locations of cortical and subcortical regions most important to relapse prediction. The middle row indicates the posterior probability of individual relapse over an observed range (minimum to maximum in 20 even increments) of region ratios locally averaged across 10 bootstrapped resamples of the data set and refitted to the derived classifier. A non-parametric LOESS model was fit to the predicted responses. Points about each line indicate predicted probabilities from each resample while rugs of each plot indicate the density of observed values in the whole sample. The bottom row illustrates corresponding distributions of random forest decision points (black) for these regions across underlying 1000 classification trees in the determination of relapse status. These are compared to distributions of these regions for relapsing (red) and non-relapsing (blue) patients

**Fig. 3:**
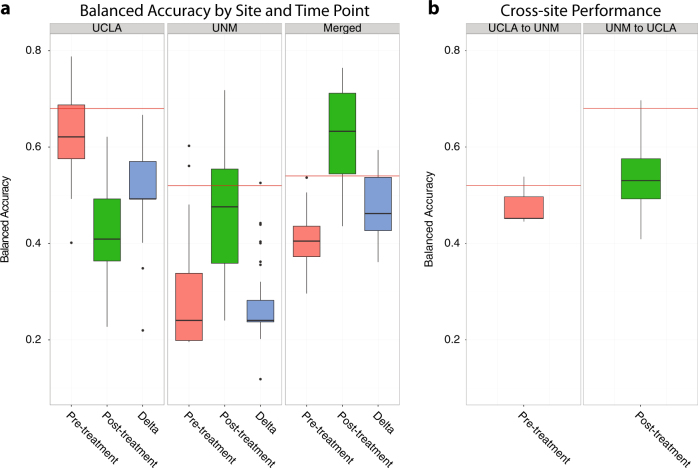
Distributions of balanced accuracies across all classifier parameterizations **a** Performances of classifiers trained and tested within a cohort. Horizontal red lines indicate the site-specific baseline detection rate. **b** Distributions of balanced accuracies achieved in cross-site predictions. All models exceeding their respective baseline detection rates within site for UCLA at pre-treatment (**b**, left) and UNM post-treatment (**b**, right) were used to predict relapse in the independent site. These performances are compared to the independent site’s respective baseline detection rate shown in the horizontal red line

Here we detail the highest performing model from each location provided their BAs are above the BDR of the cohort used to train the model. Classifier performances from pre-, post-, and change during ECT series for individual and combined sites are presented in Table 2. Regions important to each classifier are illustrated in Fig. 2. In Fig. 3 we plot the BA obtained across all parameterizations of the models explored in the grid search and compare this distribution to the model’s BDR.

**Table 2:** Classifier performance summaries: highest-performance models by site and time point

**Location**	**Time point**	**Accuracy**	**Balanced Accuracy**	**Sensitivity**	**Specificity**	**PPV**	**NPV**	**BDR (Balanced Accuracy)**
UCLA	T1	82%	78%	90%	66%	83%	80%	68%(10)
	T2	70%	62%	90%	33%	71%	66%	68% (-6)
	T∆	76%	66%	100%	33%	73%	100%	68 (-2)
UNM	T1	60%	60%	66%	53%	57%	63%	52% (8)
	T2	72%	71%	66%	76%	72%	71%	52% (19)
	T∆	52%	52%	66%	38%	50%	55%	52 (0)
Merged	T1	54%	53%	65%	42%	57%	50%	54% (-1)
	T2	76%	76%	73%	78%	80%	71%	54% (22)
	T∆	59%	59%	60%	57%	63%	55%	54% (5)

T1 Time point 1 (pretreatment), T2 Time point 2 (post-treatment), T∆ features derived from the change in values over ECT index, i.e., T2 - T1, PPV positive predictive value, NPV negative predictive value, BDR baseline detection rate. ‘Positive’ class is non-relapse. Balanced accuracy–BDR is a proxy for the improvement of the classifier above baseline chance levels of classification.

Relapse at UCLA was well-predicted from pre-treatment measures with a BA of 78%; well above the 68% BDR. Cingulate isthmus thickness asymmetry was the most important feature, followed by the ratio of left superior frontal to left rostral middle frontal cortical thickness. In post-hoc examination of these measures, relapsing patients were shown to have numerically smaller, though not statistically different, right to left hemisphere cingulate isthmus thickness ratios than non-relapsers. The asymmetry of the cingulate isthmus trended towards a significant association with sex (*t* = 2.075, *p* = 0.056, Supplementary Fig. 4). No statistically significant post-hoc relapse/non-relapser difference for the left superior frontal to left rostral middle frontal cortical thickness ratio was observed. While several UCLA-based models exceeded the BDR, many did not, suggesting some degree of model instability. Supplementary Table 1 further outlines important features that were selected by each classifier in at least 50% of LOO-CV folds.

Using RFs fitted to 10 randomly resampled sets of observations we modeled the predicted probability of a simulated patient’s relapse given an observed range of these two ratios. Each simulated patient was assigned the within-cohort average of each feature except for the ratio measure to be evaluated, which itself took on a series of 20 evenly spaced values between the observed minimum and maximum of the cohort. Predictions of the simulated patient’s outcome from each of the 10 RFs were estimated using a nonparametric LOESS model (Fig. 2a). Patients with smaller right to left cingulate isthmus ratios had lower predicted probabilities of relapse. Perturbations of the left superior frontal to left rostral middle frontal ratio exerted less influence on the posterior probability of relapse after accounting for this measure. Characteristics of the distribution of RF split points for the most important features are reported in Supplementary Results.

We assessed the generalizability of all UCLA pretreatment model parameterizations that yielded a BA greater than the UCLA BDR by attempting to predict relapse at UNM from pretreatment measures using the models trained on UCLA data (see Supplementary methods). Figure 3b illustrates the distribution of these cross-site prediction performances. The mean BA obtained by predicting relapse at UNM from UCLA models was 47% (SD = 0.03%, range = 44–53%), less than the UNM BDR of 52% and the averages of the two region ratios were not consistently larger or smaller by relapse status across sites (Supplementary Fig. 3).

Using the UNM cohort to train our classifier yielded a BA of 60%, which is slightly above the 52% UNM BDR. In addition, the highest-performing RF obtained by merging the UCLA and UNM cohorts performed at near-chance levels and below the BDR (BA = 53%, BDR = 54%).

### Prediction from post-treatment measures

Prediction of relapse at UCLA using post-treatment measures resulted in a 62% BA, below the 68% BDR. In contrast, post-treatment measures were the best predictors of relapse at UNM yielding a BA = 71%, well above the 52% BDR. Here, the right to left pallidum volume ratio was the most discriminative feature. Groupwise means of this feature did not differ (*t* = −0.21, *p* = 0.82). The second most informative feature was left pericalcarine to right precuneus cortical thickness ratio, which tended (non-significantly) to be larger among non-relapsing patients (*t* = −1.94; *p* = 0.06). Using the fitted RF to predict simulated patients, we observed that predicted relapse was lowest when the right to left pallidum volume ratio was ~0.9 and the left pericalcarine to right precuneus ratio was ~0.7, but increased steadily as these ratios deviated from these points (Fig. 2b).

RFs with BAs above the UNM BDR generally performed poorly when used to predict relapse at UCLA from post-treatment measures attaining an average BA = 54% (SD = 0.06, range = 40–69%) with most falling below the UCLA BDR of 68% (see Fig. 3b). Prediction of relapse at UCLA using post-treatment measures resulted in a BA of only 62%, below the BDR.

Classification using the combined cohorts resulted in a BA of 76% using post-treatment measures. The left lateral occipital lobe to left pericalcarine gyrus (*t* = 3.75, *p* = 0.0005) and left paracentral gyrus to left precentral gyrus (*t* = 2.46, *p* = 0.019) ratios were most informative with both ratios being significantly larger in relapsers. Patients with larger ratios had greater a predicted probability of relapsing (Fig. 2c). Interestingly, the majority of this model’s parameterizations were well above the BDR suggesting it is relatively stable across parameterizations (Fig. 3).

### Prediction from measures of change

When examining pre to post ECT-related change in structural imaging markers, both sites did not show predictive utility above their respective BDRs (UCLA = 66% and UNM = 52%). Merging the two sites resulted in a BA of 59% only slightly above the BDR of 54%.

## Discussion

To our knowledge this is the first study to predict relapse/recurrence of depression following ECT where findings are expected to be relevant to prediction of other antidepressant treatments. We used advanced classification algorithms and novel feature representations including structural neuroimaging, demographic, and clinical measures to identify biomarkers of long-term clinical outcome. Our investigation extends beyond many classification-based studies by further interrogating our fitted models to understand the relationships between salient features and a patient’s relapse risk. This is an important step in the application of machine learning to data with clinical implications since a black-box algorithm yield less information about the driving mechanisms. Using these tools, we demonstrated that ratios and asymmetries of particular cortical and subcortical brain regions implicated in the pathophysiology of depression present promising biomarkers for prognosis of symptom recurrence and/or relapse.

### Significance of intra and interhemispheric regional ratios

Remarkably, using an entirely data-driven approach, we identified that the intra and interhemispheric ratios of homologous or proximal regions present biomarkers of symptom recurrence/relapse in MDD. The validity of these findings is bolstered by the observation that the selected ratios do not appear to be randomly distributed throughout the brain as we would expect if we were merely detecting noise. Instead, the constituents of these ratios were either homologous or neighboring regions.

A substantial body of evidence suggests asymmetrical neural representation of emotional control and processing^31,32^ and structural and functional imaging findings commonly report lateralized effects in MDD^33–35^. Several previous studies have related hemispheric asymmetry to clinical outcome following antidepressant treatment. For example, differential patterns of asymmetric functional connectivity have been noted between rTMS responders and non-responders^36^. The intracranial distribution of the electric field induced by ECT, and therefore the set of structures most affected, is shown to be impacted by electrode placement^37^. Since both our sites used predominantly right unilateral electrode placement, regional asymmetries and/or ratios observed post-treatment may affect clinical outcome.

### Regions influencing relapse risk

The highest performing model was obtained when the cohorts were combined. Nonetheless within-site models warrant discussion due to the significant age differences between the cohorts. ECT has been established to have greater clinical benefits in older individuals^38^, which suggests that changes in neural integrity with age might modulate the antidepressant mechanisms of ECT. Thus differential sets of important features across site may highlight age-related predictors of relapse risk.

Within the UCLA cohort we observed that the hemispheric balance of cingulate isthmus thickness and the ratio of left superior to left rostral middle frontal cortical thickness were highly predictive of relapse from pre-treatment ECT measures. Cingulate isthmus asymmetry was additionally associated with sex (Supplementary Fig. 4), which suggests sex is a potentially informative predictor of relapse. Women are known to be at a two-fold increased lifetime risk for MDD compared to men^39^. This trend is reflected both by the higher proportions of women and the higher relapse rates observed in women at both our study sites. Structural and functional abnormalities of the cingulate are repeatedly implicated in depression^40,41^. Notably, prior studies assessing imaging markers of longer-term outcomes have shown links with pre-treatment cingulate volumes^12,13^. This region forms a key part of the limbic system involved in memory and other complex cognitive functions^42^. Our UCLA-based model also identified the ratio of the left superior to left rostral middle frontal cortical thickness as important. This feature may point to altered reward and mood regulation circuitry, which include dorsolateral prefrontal nodes. The involvement of these regions is consistent with widespread reports of fronto–striatal–limbic network disruptions in MDD^43–45^.

Post-treatment measures best informed relapse prediction at UNM. The asymmetry of pallidum volume and the left pericalcarine to right precuneus cortical thickness ratio was critical to prediction. The pericalcarine and precuneus are cortical association regions in close proximity and reciprocally connected to the retrosplenial cingulate observed in the UCLA cohort. Notably, the precuneus forms part of the default mode network consistently implicated in MDD^46^. In a sample overlapping with this study, our group recently reported on differential ECT-induced morphological changes in the pallidum between responding and non-responding MDD patients following ECT^47^. The current findings suggest these regions are also relevant to relapse. Structural pallidal abnormalities are repeatedly implicated in MDD pathology and again point to disruptions in fronto–striatal–limbic circuitry though at different network nodes^48,49^. Further, depressive symptomatology including amotivation, anhedonia, apathy, and rumination are often linked to abnormalities of the ventral striatum and pallidum^50–52^, suggesting a potential functional correlate.

Within-site classifiers were additionally tested by attempting to predict relapse at the held-out site. These classifiers’ BAs were below the independent sites’ BDRs. These may result from systematic differences across the two cohorts. Principally, UNM patients were significantly older than UCLA patients and only the UCLA cohort was tapered from medications during the acute phase of ECT treatment. Age-related neurodegeneration well-established^53,54^ and interactions with depression may present confounds for classifiers trained on patients with differing ages. Further, late onset depression may have a different etiologies such as vascular contributions from cardiovascular disease and hypertension^55,56^ that could predispose geriatric patients to depressive symptoms. Illness duration and lifetime number of depressive episodes are also likely to be greater in older individuals may impact symptom recurrence^8^. Given these systematic differences, future work will validate these models on well-matched independent cohorts.

The highest performing model, in terms of BA above its BDR, was obtained using post-treatment information from the combined cohorts resulting in BAs consistently above the BDR across nearly every model parameterization, whereas site-specific classifiers inconsistently outperformed the BDR. Here, the ratios of two pairs of ipsilateral and neighboring left hemisphere cortical regions were most informative in relapse prediction: the paracentral to precentral cortical thickness ratio and the lateral occipital to pericalcarine cortical thickness ratio. Paracentral thickness has been linked with impulsivity in MDD^57^ and emotional perception and interpretation^58^ A recent review of 10 studies surveying 329 first-episode patients and 340 healthy controls also reported a consistent increase in the left paracentral lobe volume^43^. Several studies have also reported change in motor circuits in relation to MDD^59^. Studies using transcranial magnetic stimulation^60^ and ECT^61^ have specifically shown significant changes in left lateralized motor-related cortical excitability, suggesting a mechanism of treatment response, which may also impact relapse.

The second highly predictive feature in the pooled classifier was the left lateral occipital to left pericalcarine cortical thickness ratio. White matter microstructural, resting state connectivity, and volumetric abnormalities in MDD have been noted in these regions^62–65^. In a study of 65 patients with recurrent MDD and 65 matched controls, Na et al. identified significantly thinner lateral occipital lobes in MDD patients versus controls^66^. The pericalcarine is arguably less studied or implicated in MDD. However, prior studies have shown changes in gamma-aminobutyric acid within this region in relation to different antidepressant treatments, including ECT^67–69^.

In the context of prior reports, the constituents of the regional ratios informing our classifiers have plausible biological grounding. Depression is considered a brain-network disorder^40^, thus structural abnormalities are expected and reported to occur in spatially diffuse regions. As such, a data-driven approach capturing the relative thickness/volume relationships of spatially distinct regions is valuable. As a brain-network disorder, ECT-induced neural plasticity of these spatially diffuse regions is a plausible mechanism by which ECT may mediate relapse. A body of pre-clinical and neuroimaging data suggest that changes in neural plasticity may contribute to the antidepressant effects of ECT^70,71^. Symptom recurrence may likewise relate to neuroplastic processes which vary across brain regions and may return to pre- or post-ECT homeostasis to predict future recurrence. Though this interpretation remains speculative, our results support that structural variations in particular brain features before and after treatment impact future relapse.

It is noteworthy that post-treatment measures yielded more accurate predictions for both UNM and the merged cohorts than pre-treatment or change measures. One plausible reason for this is that changes in brain morphometry induced by ECT are highly related to relapse and this information is unavailable at pre-treatment. However, we might expect change measures to be more informative than either time point in isolation. But, from a statistical perspective, change measures include noise from both time points, which is not proportionally offset by differing signals from pre- and post-treatment measures.

### Limitations

While this is the first study to attempt prediction of depression relapse following ECT, there are important limitations. It was only possible to determine predictors of relapse in patients initially showing treatment response where the UCLA and UNM cohorts consisted of 17 and 25 ECT responders, respectively. As models using the combined cohorts consistently outperformed the BDR, while models from individual sites did not, within-site models were likely underpowered. To maximize the number of participants used to train our classifiers, we used leave-one-out cross-validation. A related limitation is that since the highest performing model was built using the merged cohorts we cannot evaluate its generalizability.

In addition, several patients transitioned from RUL to BL electrode placement during ECT index. Because BL arrangement is associated with higher remission/response rates it is also possibly associated with differential relapse rates relative to RUL. However, only a minority of patients were transitioned: Four at UNM (three of whom relapsed) and one at UCLA (a non-relapser). The small number of transitioned patients precludes statistical associations of relapse and transition status.

Differences in medication status by site is noteworthy. The UCLA cohort was tapered off of psychotropic medication before ECT unlike UNM patients. Several studies have noted that ECT increases the permeability of the blood-brain barrier^72,73^ promoting interactions between ECT and medication. Such an interaction could augment clinical outcomes between sites.

The use of m-/c-ECT is also potentially meaningful. Although we observed no association between m-/c-ECT status and relapse rates in either site we did not include m-/c-ECT status as a predictor due to its ostensible lack of influence and because it is an unknown factor at each time point in practice.

Though the poor generalizability of the classifiers trained within site is possibly due to overfitting and/or small sample sizes, biological differences between elderly and non-elderly patients may be relevant. Since the highest performing models were derived from pooling the cohorts we anticipate improved generalizability with larger samples. Further, we acknowledge that our definition of relapse as an initially responsive patient (i.e., reduction of HAM-D by ≥ 50%) and a subsequent return to a HAM-D ≥ 17 is one of several possible definitions, to avoid a potential increase in type 1 errors we did not attempt classification of relapse according to other definitions.

A further criticism is the use of a grid-search over a large parameter space whereby one may expect to identify models exceeding the BDR merely by chance. However, rather than viewing each parameterization of the model as a separate model, the identification of a set of parameters maximizing the model’s performance can simply be viewed as the optimization of a single model. If, however, each reparameterization of the model is viewed as an independent model, the proportion of parameterizations yielding performances above chance can be evaluated statistically. This latter view is explored in Supplementary Results Section.

## Conclusions

Though much work remains to develop models with cross-site predictive value, our current findings are novel and add important new leads. For each site we identified models that performed well above their respective BDRs. Currently, post-treatment measures appear most predictive. Models based on pretreatment measures might be useful for clinical decisions regarding patient exposure to the unwanted side-effects of ECT if benefits are not enduring. However, post-treatment models, which yielded BAs between 71 and 78%, may contribute towards more targeted monitoring of patients at elevated risk of relapse allowing for more timely prevention strategies.

## Electronic supplementary material

Supplementary methods and results

Supplemental figure 1

Supplemental figure 2

Supplemental figure 3

Supplemental figure 4
