# Supplementary material for: Inter and intra-hemispheric structural imaging markers predict depression relapse after electroconvulsive therapy: a multisite study
Source: Transl Psychiatry. 2017 Dec 8;7:1270. doi: 10.1038/s41398-017-0020-7 (PMC5802464; doi:10.1038/s41398-017-0020-7)
Supplement: Supplementary file 1 — Supplementary methods and results [file 41398_2017_20_MOESM1_ESM.docx]

**Supplementary methods**

*Random forest tuning parameters*

Random forests (RF) were tuned over a space of *mtry* values, the number of randomly selected features passed to each classification and regression tree (CART) node, but *sampsize* (the number of boostrapped samples redrawn for each CART), *nodesize* (the minimum size of terminal nodes), and *maxnodes* (the allowable depth of each tree) were all left to their default values: sampsize=63.2% of observations, nodesize=1, and *maxnodes*=null (i.e., trees are grown to their full, unpruned extent). 1000 trees composed each RF and specificity was optimized because numerous solutions were available that maximized sensitivity or accuracy at the expense of low specificity.

*Classifier profiling*

Many studies involving machine learning, regardless of the underlying classifier, treat the classifier as a black box reporting only the accuracy of the classifier. In certain settings this is sufficient, however, for clinical applications it is beneficial to understand how certain features contribute to the predicted probability of a patient’s class. We contribute two novel steps towards unveiling the relationships between values of certain features and the probability that a patient will relapse. For the most important feature(s) in the reported models we used RFs fit to 10 random resamples of the given set of observations to predict the probability of a hypothetical patient’s relapse. The hypothetical patient’s features were all fixed to observed averages while the value of the most important feature(s) took on a range of observed values (taken in 20 even increments from the observed minimum to maximum). Non-parametric LOESS models were then fit to the predicted probabilities of relapse to visualize the predicted probability of an individual’s relapse given a plausible range of values for the most important feature(s). We additionally used kernel density estimation to plot the distributions of forest-wise split points for the most important feature(s). The peak of these distributions are reported in order to understand the critical values most indicative of class differences (see Figure 2).

*Cross-site predictions*

For convenience in referring to multiple data sets arising from two independent sites, we here refer to the data set a model was initially trained on as the *source data* and the independent data set the model later attempts to classify as the *target data*.

All models across all grid search parameterizations having BAs greater than the source site’s BDR were further evaluated by attempting to predict relapse at the target site using features from the same time point. Because the set of features used to predict held-out observations in the source data varies across each fold there is no clearly defined set of variables identified as optimal to predict cases in the target data. We therefore allowed the frequency of a feature’s selection in the source data to be a parameter in the process of feature set selection for models used to predict target data. Features selected in the source data below a percentage threshold *p* $\in$ {0%, 10%, 20%,…, 100%} were excluded. A random forest was then trained on the source data set using the remaining set of features and optimized to maximize overall specificity across 10-fold cross-validation. The resultant model was then used to predict relapse status in the whole target data set.

*Classification with a rejection option*

In certain domains classification should only be attempted when the posterior probability of class assignment is sufficiently high, that is, when the classifier is confident in its prediction. Observations about which the classifier is uncertain may be referred to a domain expert for further review to reduce errors. This is known as adopting a rejection option^70^ and has previously been applied to classification of clinical outcome in depressed patients by Schmaal and colleagues^14^. Here we explore how the accuracy of our classifiers change as a function of classification using only patients with classification confidence above a smoothly varying threshold which results in what is known as an accuracy-rejection curve, introduced by Nadeem^27^. We define confidence as *1 - min{p(relapse), p(non-relapse)}* where *p* is the posterior probability of each class and plot the accuracy as a function of the confidence threshold.

**Supplementary Results**

*Distributions of random forest split points*

We assessed the distribution of split points across all 1000 underlying classification trees composing the optimized random forest (RF) to gauge which ratio values were indicative of subsequent relapse for the top performing models at each site (Figure 2, bottom row). For the right to left cingulate isthmus ratio, important for predicting relapse at UCLA from pretreatment measures, the peak of the gaussian kernel density distribution derived from these split points was 0.94, and 78% of these split points were within 1 standard deviation (SD=0.04) of this peak suggesting that this is a critical decision point (Figure 2a). The peak of the distribution for left superior frontal to left rostral middle frontal ratio split points was 1.13, and 67% of splits occurred within 1 standard deviation (SD=0.04).

In the UNM cohort, relapse was best predicted from post-treatment measures. Assessing the distribution of RF split points here showed that the peak of the distribution of classification tree split points for the pallidal ratio was 0.95, and 71% of split points were within 1 standard deviation (SD=0.09). The mode of the second ratio was 0.67, and 70% of split points were within 1 standard deviation (SD=0.04).

Post-treatment measures were again the most informative of relapse when the two cohorts were merged. Evaluation of the RF split points for the most important features showed that the peak for the left lateral occipital lobe to left pericalcarine gyrus ratio was 1.46, and 64% of split points fell within 1 standard deviation (SD=0.05). Meanwhile the peak of split points for the left paracentral gyrus to left precentral gyrus was 0.95 with 63% of the splits falling within 1 standard deviation (SD=0.039).

*Classification performance with a rejection option*

In Supplementary Figure 1 we illustrate the overall accuracy, rather than the BA, and corresponding sensitivities and specificities of each classifier obtained when only subjects with a posterior class probability above some level of confidence are classified. For the UCLA pretreatment model a minimum confidence of 61% yielded 100% accuracy while the required confidence at UNM was 89% for 100% accuracy. Interestingly, when the cohorts were combined, increasing minimum levels of confidence did not appear to benefit the classifier’s performance. Additionally, at higher levels of confidence approaching 100%, specificity was very low. Pursuant to this, 17 patients from the merged group were assigned a confidence level above 90% yet 4 of these patients, 2 from each class, were misclassified.

*Post-hoc analyses with repeated 10-fold cross validation*

We repeated the classification analysis for each time point and location using 10 repeated 10-fold cross-validation rather than LOO-CV in order to gauge our certainty about our highest performing models. Here, rather than holding a single patient out at each iteration, approximately 1/10^th^ are held out for subsequent validation. This process was repeated 10 times with patient assignment to each fold being randomly reassigned at each repetition.

The UCLA model using pretreatment features with repeated 10-fold cross-validation achieved an average BA of 70% (range= 62-78%) across all folds where 4/10 folds resulted in BAs below the BDR (68%). Here, the most informative features did not agree with the original LOO-CV approach and were (i) the left fusiform to right parahippocampal thickness ratio, and (ii) the left middle temporal to left rostral middle frontal thickness ratio.

At UNM the post-treatment repeated 10-fold model yielded an average BA of 58% (range=43-72%) with 3/10 folds resulting in BAs below the UNM BDR (52%). The most important features were in partial agreement with those recovered by the LOO-CV approach: (i) the right to left pallidum volume ratio, (ii) right banks of the superior temporal sulcus to right supramarginal gyrus ratio, and (iii) the left pericalcarine to right precuneus thickness ratio.

Interestingly the repeated 10-fold cross validation based on the merged cohort from post-treatment measures was still the best performer with an average BA of 70% (range=64-77%), all well above the BDR (54%). The most informative regions were again in agreement with the LOO-CV approach: (i) left lateral occipital to left pericalcarine thickness, (ii) left paracentral to left precentral thickness, and (iii) right to left pallidum volume ratios.

*Multiple comparisons and model significance*

If one views each parameterization of the model as a separate, independent model we can naively employ binomial tests to evaluate the significance of x/N models performing above chance. Theoretically N = 100, but in reality not all models were viable. Correlation thresholds (the $\left| r \right|$ parameter) below |0.3| were too strict in most cases resulting in elimination of all but one feature and thus random forests did not run. $\left| r \right|$ above 0.7 was, on average, too lenient and resulted in models that were too computationally expensive and intractable. Thus, excluding non-viable parameterizations, we were left with 50 models for UCLA at time point 1, 54 for UNM at time point 2, and 50 from the merged groups from time point 2; recall that these were the highest performing models within sites.

The proportion of models exceeding the BDR when the groups were merged using time point 2 features was significant using a binomial test: P(Y≥37 | n = 50, p = 0.5) = 0.00046. This proportion was not significant for the UNM cohort using time point 2 features: P(Y≥17 | n = 54, p = 0.5) = 0.9981. Similarly this proportion was not significant for the UCLA cohort using time point 1 features: P(Y≥13 | n = 50, p = 0.5) = 0.9998.

But, a binomial test does not take into account that the models’ performances are associated with the two parameters over which we conduct a grid search, $\left| r \right|$ and ⍴ (the quantile-based threshold for how frequently a feature was selected). The correlation between performance and parameterization may reduce the effective number of independent tests. Specifically, the partial correlation between the BA and $\left| r \right|$ while controlling for ⍴ in the merged group using time point 2 features is significant (r = -0.67; p = 9.461388e-08). That is, performance (BA) is worse at higher $\left| r \right|$ values, on average. BA is similarly significantly negatively correlated with $\left| r \right|$among the UNM group when using time point 2 features (r = -0.50; p = 0.00011). Neither site’s performance was significantly associated with ⍴ and neither parameter was significantly associated with performance within the UCLA cohort using time point 1 features.

Given that performance is significantly associated with $\left| r \right|$ in the merged and UNM cohorts we could instead evaluate whether the proportion of BAs exceeding the local BDR within each parameterization of is significant. For UNM we observed that 10/10 models parameterized under $\left| r \right|$=0.3 (the lowest viable parameterization) exceeded the BDR, yielding a significant proportion P(Y≥10 | n = 10, p = 0.5) = 0.00097. No $\left| r \right|$ parameterizations above 0.3 yielded performances significantly above the UNM BDR. For the merged cohort, 30/30 models yielded BAs > the merged BDR over the $\left| r \right|$ parameterizations [0.3 to 0.5], each resulting in significant proportions, P(Y≥10 | n = 10, p = 0.5) = 0.00097. However, $\left| r \right|$ > 0.5 was not significant.

**Supplementary Figure Legends**

**Supplementary Figure 1.** Classifier performance with a rejection option. Accuracy-rejection curves (top) and histograms of subject-wise classification confidence by true class (bottom). Accuracy-rejection curves illustrate the overall accuracy when a reject option is allowed. With a reject option, only patients with posterior class probabilities above a given threshold (confidence) are classified. Remaining patients are ‘rejected’ for further clinician review. The accuracy-rejection curves are for the (**a**) UCLA pretreatment, (**b**) UNM post-treatment, and (**c**) merged post-treatment models. Dashed curves illustrate the corresponding sensitivities (blue) and specificities (green).

**Supplementary Figure 2.** Modeling random forest split point distributions. Each classification and regression tree (CART) comprising a random forest (RF) was searched to identify nodes using the most important feature, X_i_ (red nodes) to optimally split the observations. The values of these split points were collected for each of the 1000 underlying CARTs and the distribution of these values was compared to the distributions of feature X_i_ within class A and B.

**Supplementary Figure 3.** Distributions of most important features by site. (**a**) The left superior frontal to left rostral middle frontal cortical thickness ratio did not differ significantly by relapse status at either site. This ratio was (non-significantly) larger among non-relapsing patients at UCLA but not at UNM. (**b**) The right to left hemisphere isthmus cingulate cortical thickness ratio was non-significantly smaller among relapsing versus non-relapsing patients at UCLA on average. In contrast, this ratio was larger on average among relapsing versus non-relapsing patients at UNM though this was again a non-significant difference. (**c**) The right to left pallidal volume ratio was larger among relapsing patients across both sites on average though the difference is not statistically significant. (**d**) The left pericalcarine to right precuneus thickness ratio was larger in non-relapsing patients across both sites on average, however, the difference was non-significant at both sites. (**e**) The left paracentral to left precentral gyrus thickness ratio was larger on average among relapsing patients across both sites, however, this was a non-significant difference. (**f**) The ratio of the left lateral occipital to left pericalcarine cortical thickness was significantly larger among relapsing patients across both sites at post-treatment.

**Supplementary Figure 4.** Distribution of most important features by sex. (**a**) Left superior frontal to left rostral middle frontal cortical thickness ratio (**b**) right to left isthmus cingulate thickness ratio, (**c**) right to left pallidum volume ratio, (**d**) left pericalcarine to right precuneus thickness ratio, (**e**) left paracentral to left precentral cortical thickness ratio, and (**f**) left lateral occipital to left pericalcarine cortical thickness ratio. The right to left isthmus cingulate thickness ratio in (**b**) trended towards a significant sex-based difference (p=0.056) suggesting a possible confound of sex when using this feature for prediction. No other features were significantly associated with sex.

**Supplementary Tables**

| Supplementary Table 1. Important features by model and their selection frequencies | | |
| --- | --- | --- |
| **Model** | **Feature** | **Selection Frequency** |
| UCLA, Pretreatment | Right cingulate isthmus to left cingulate isthmus cortical thickness ratio | 64% |
|  | Left superior frontal to left rostral middle frontal cortical thickness ratio | 64% |
|  | Left superior parietal to right inferior temporal cortical thickness ratio | 58% |
| UNM, Post-treatment | Right pallidum to left pallidum volume ratio | 100% |
|  | Left pericalcarine to right precuneus cortical thickness ratio | 64% |
|  | Right precuneus to left pericalcarine cortical thickness ratio | 52% |
|  | Right banks of the superior temporal sulcus to right supramarginal cortical thickness ratio | 52% |
| Merged, Post-treatment | Left paracentral to left precentral cortical thickness ratio | 83% |
|  | Left lateral occipital to left pericalcarine cortical thickness ratio | 80% |
| Here we define features as important only if they were selected in over 50% of the internal feature selection iterations. Selection frequency is defined as the proportion of times a feature was selected across all leave-one-out cross validation folds. | | |
