# Supplementary figures and images for: Inter and intra-hemispheric structural imaging markers predict depression relapse after electroconvulsive therapy: a multisite study
Source: Transl Psychiatry. 2017 Dec 8;7:1270. doi: 10.1038/s41398-017-0020-7 (PMC5802464; doi:10.1038/s41398-017-0020-7)

**a** UCLA Pre-treatment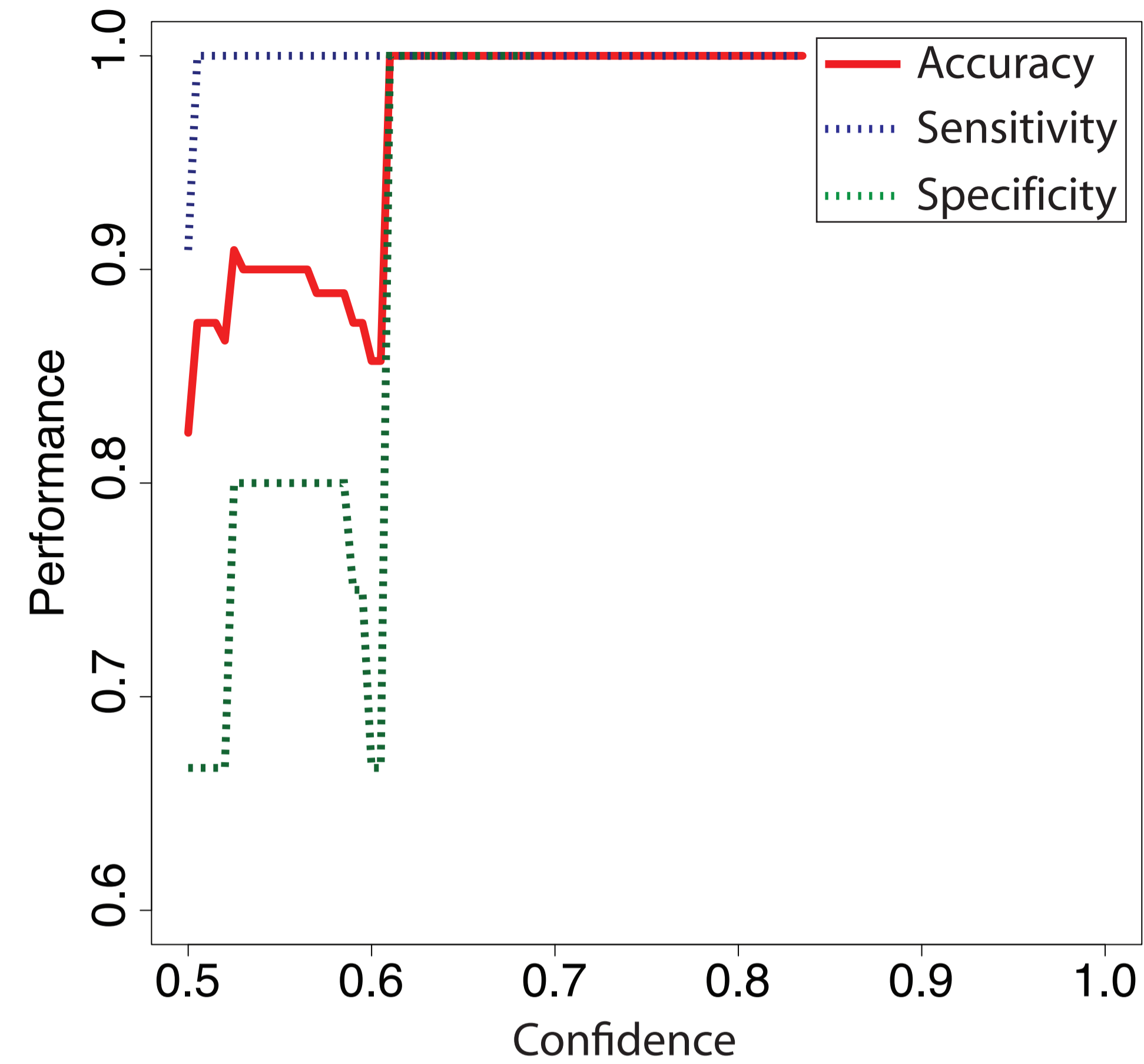**b** UNM Post-treatment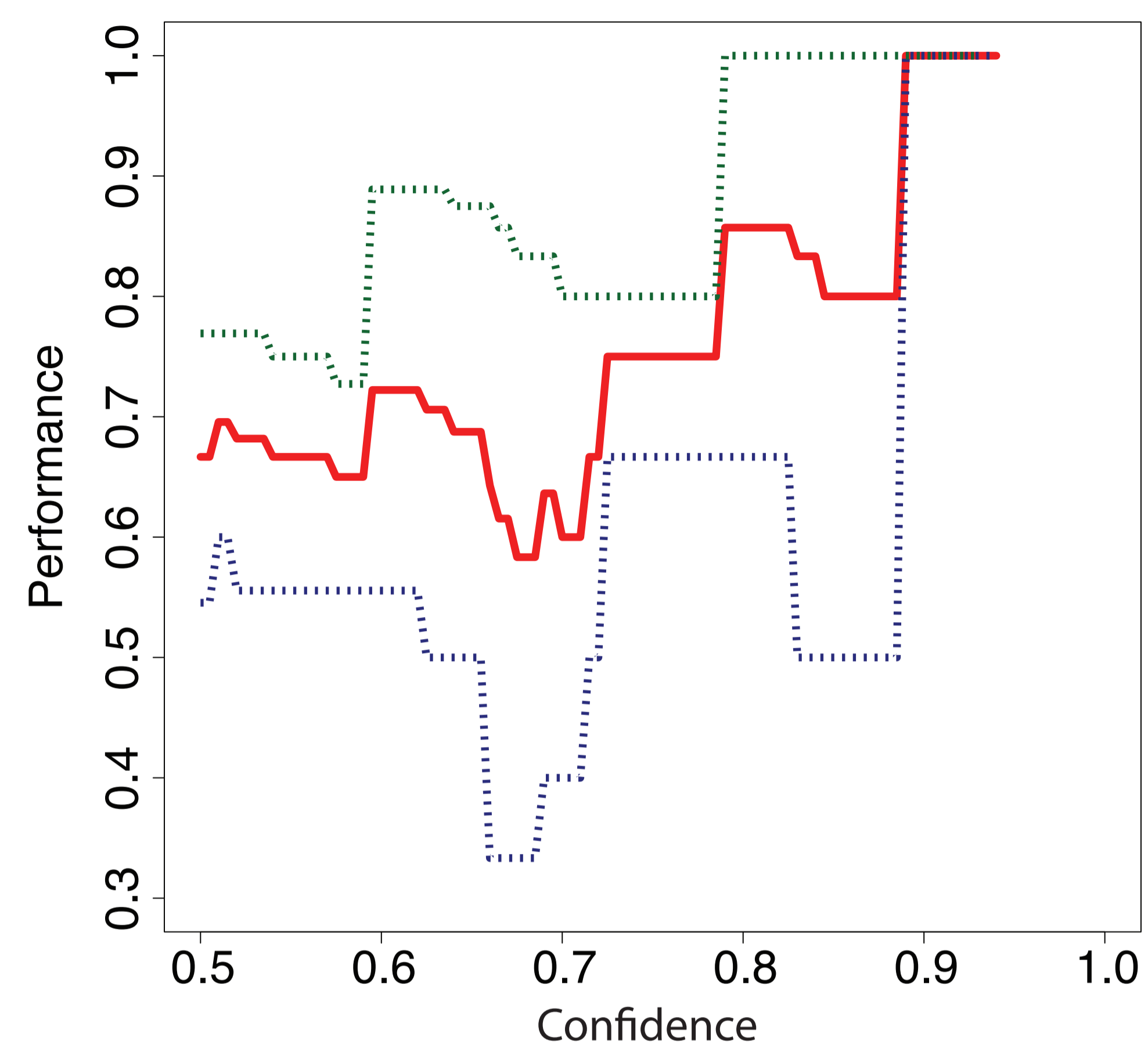**c** Merged Post-treatment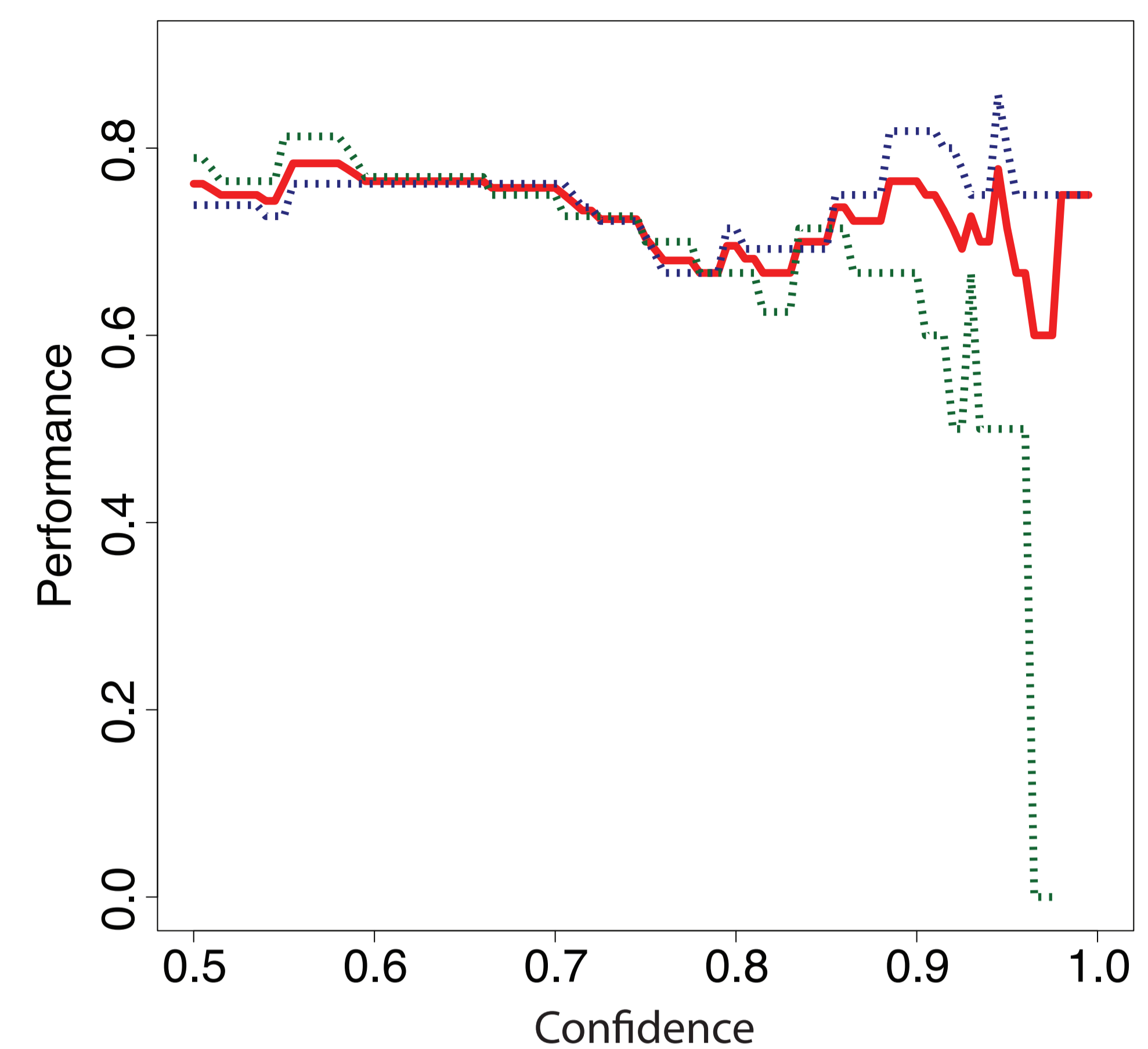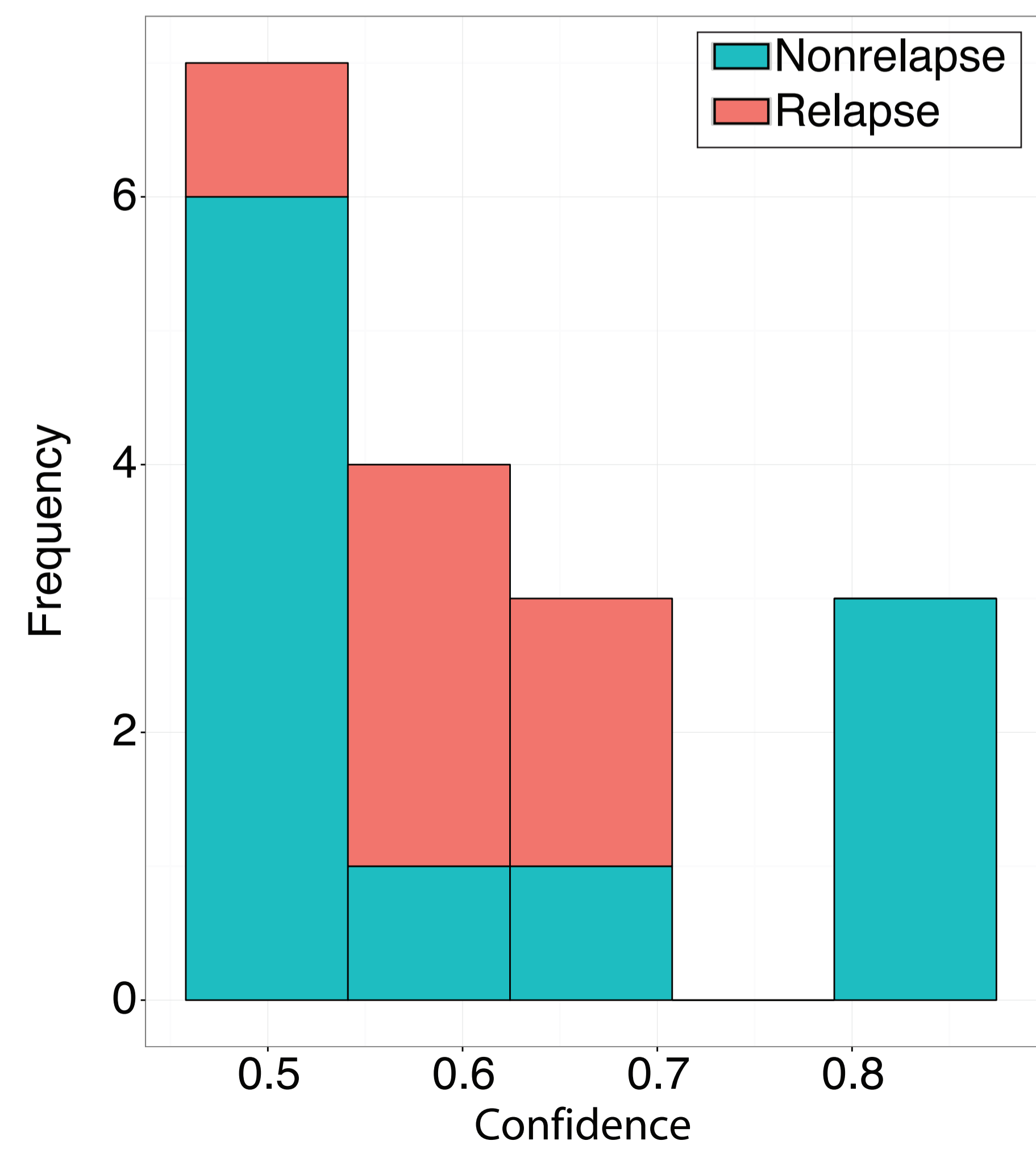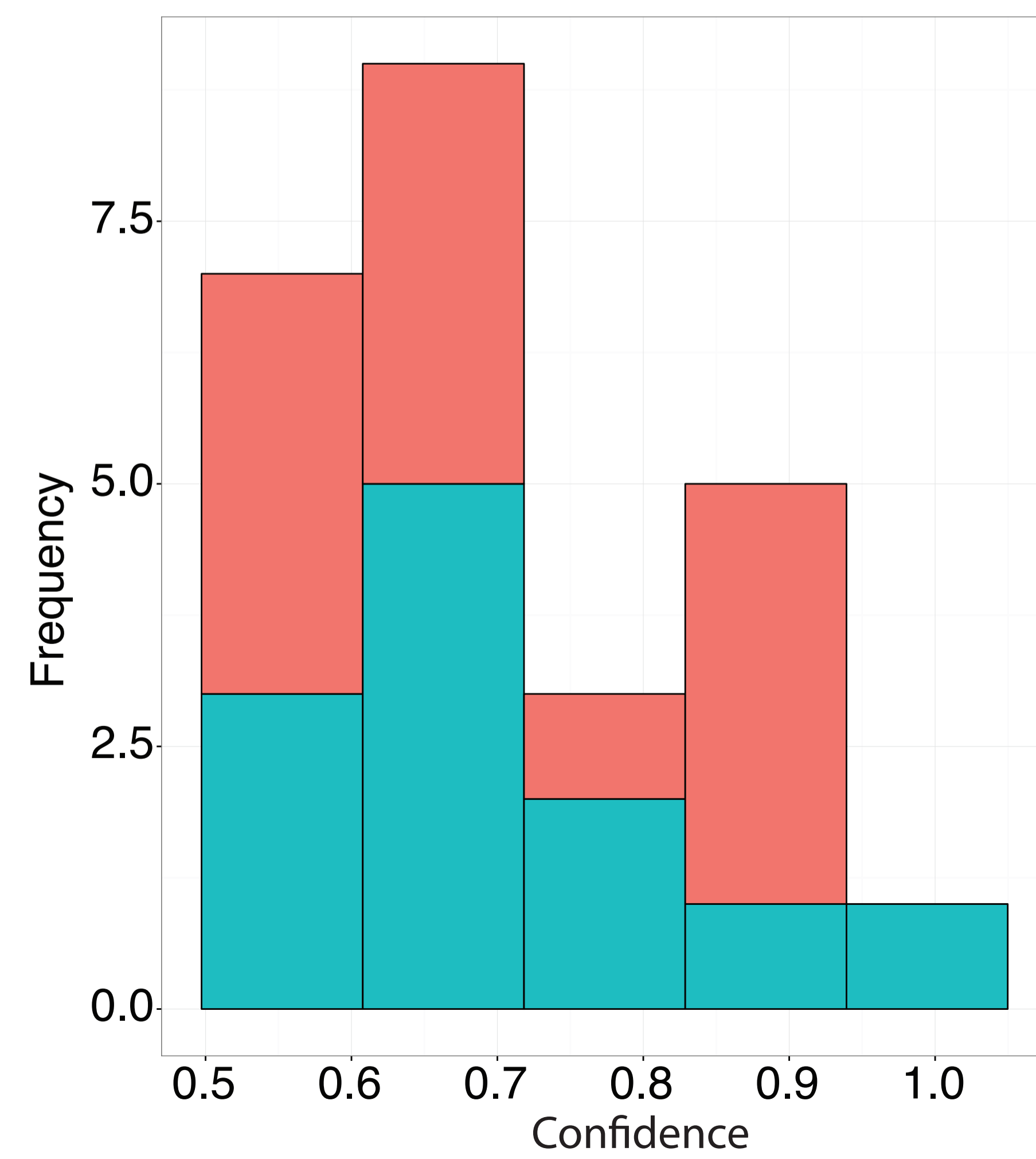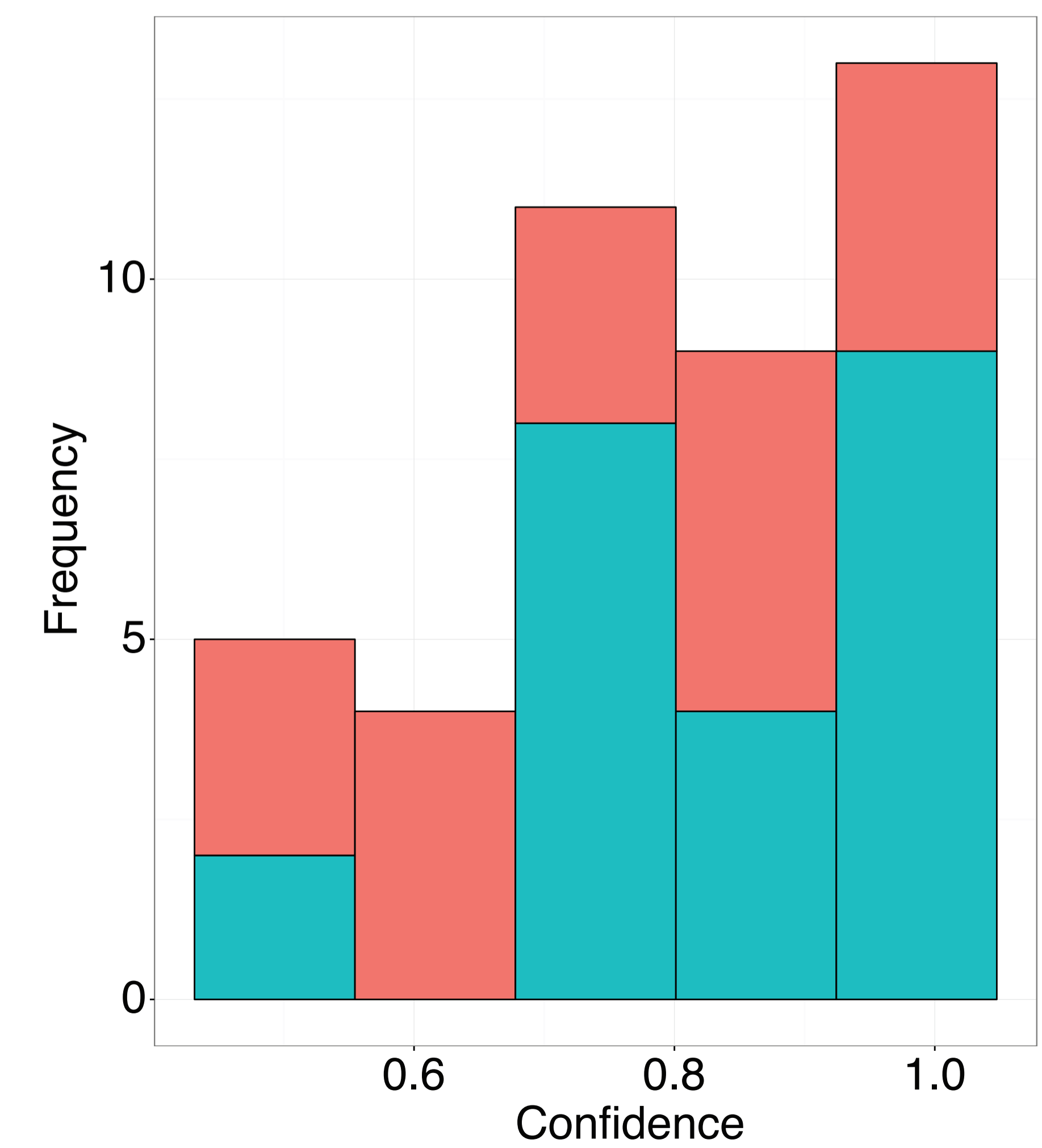

Supplement: Supplementary file 2 — Supplemental figure 1 [file 41398_2017_20_MOESM2_ESM.pdf]

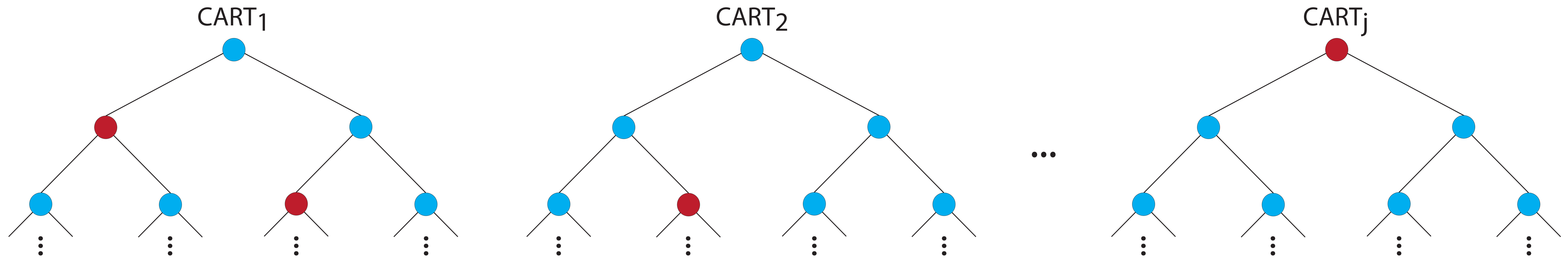

$S = \{s_1, s_2, \dots, s_n\}$  = set of split points  
for feature  $X_i$  at node  $k$  in  $CART_j$

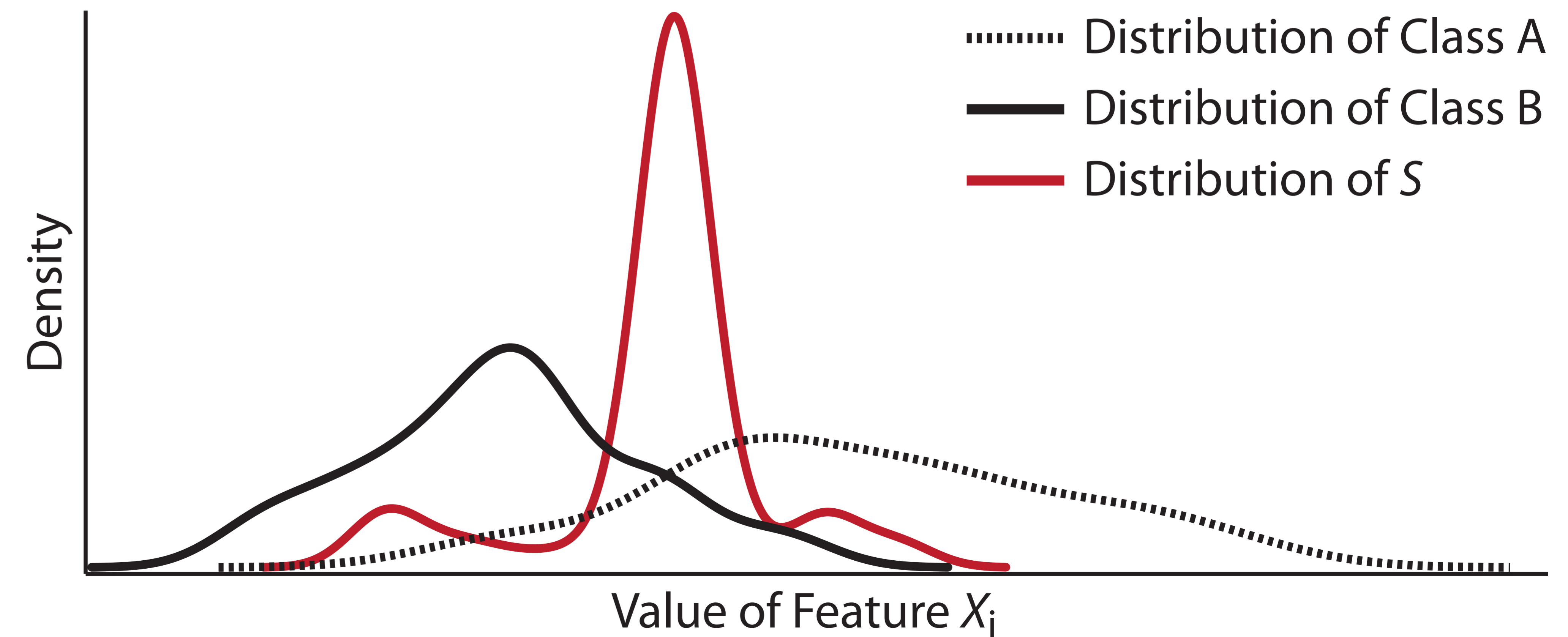

Supplement: Supplementary file 3 — Supplemental figure 2 [file 41398_2017_20_MOESM3_ESM.pdf]

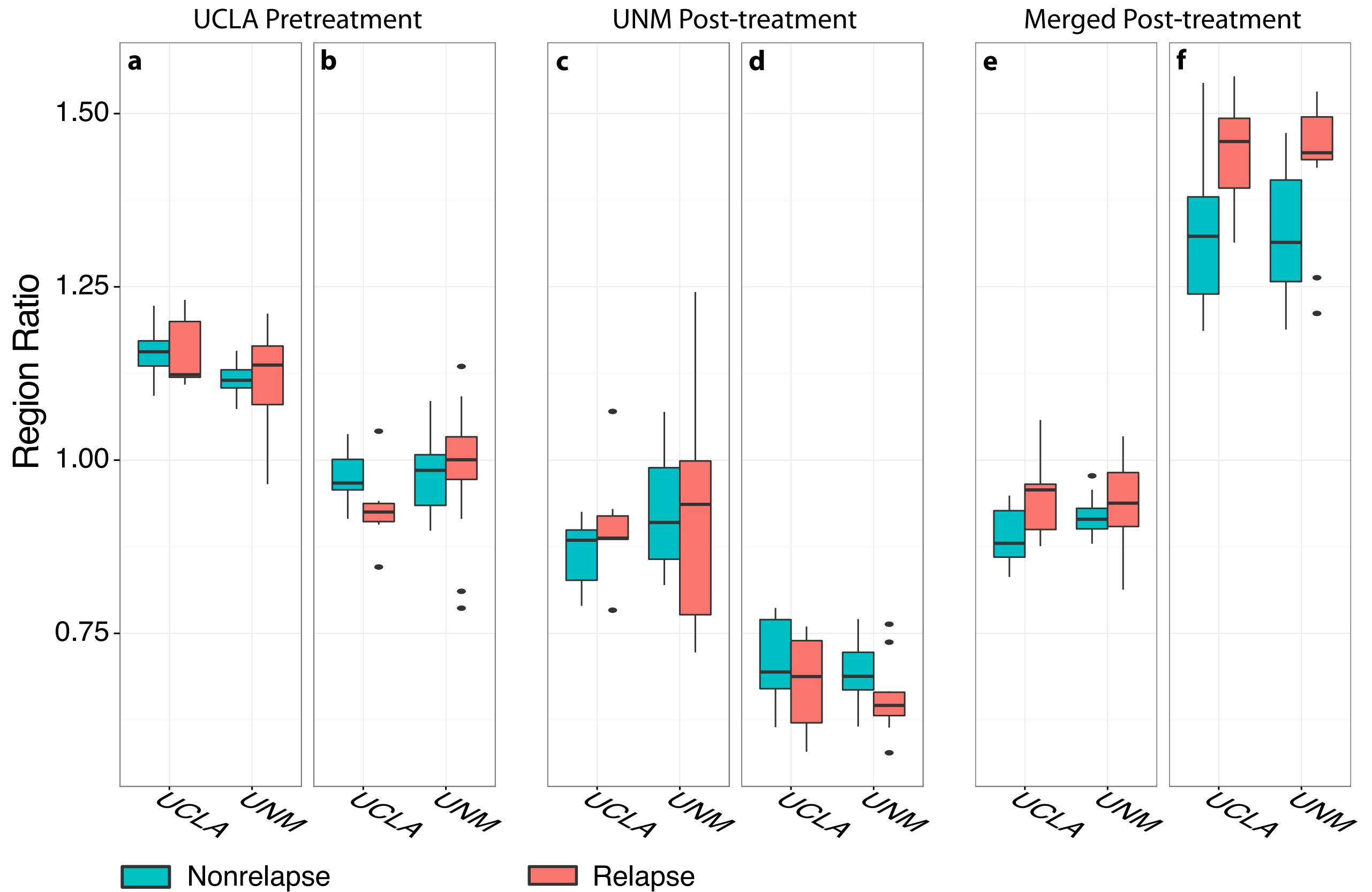

Supplement: Supplementary file 4 — Supplemental figure 3 [file 41398_2017_20_MOESM4_ESM.pdf]

UCLA Pretreatment

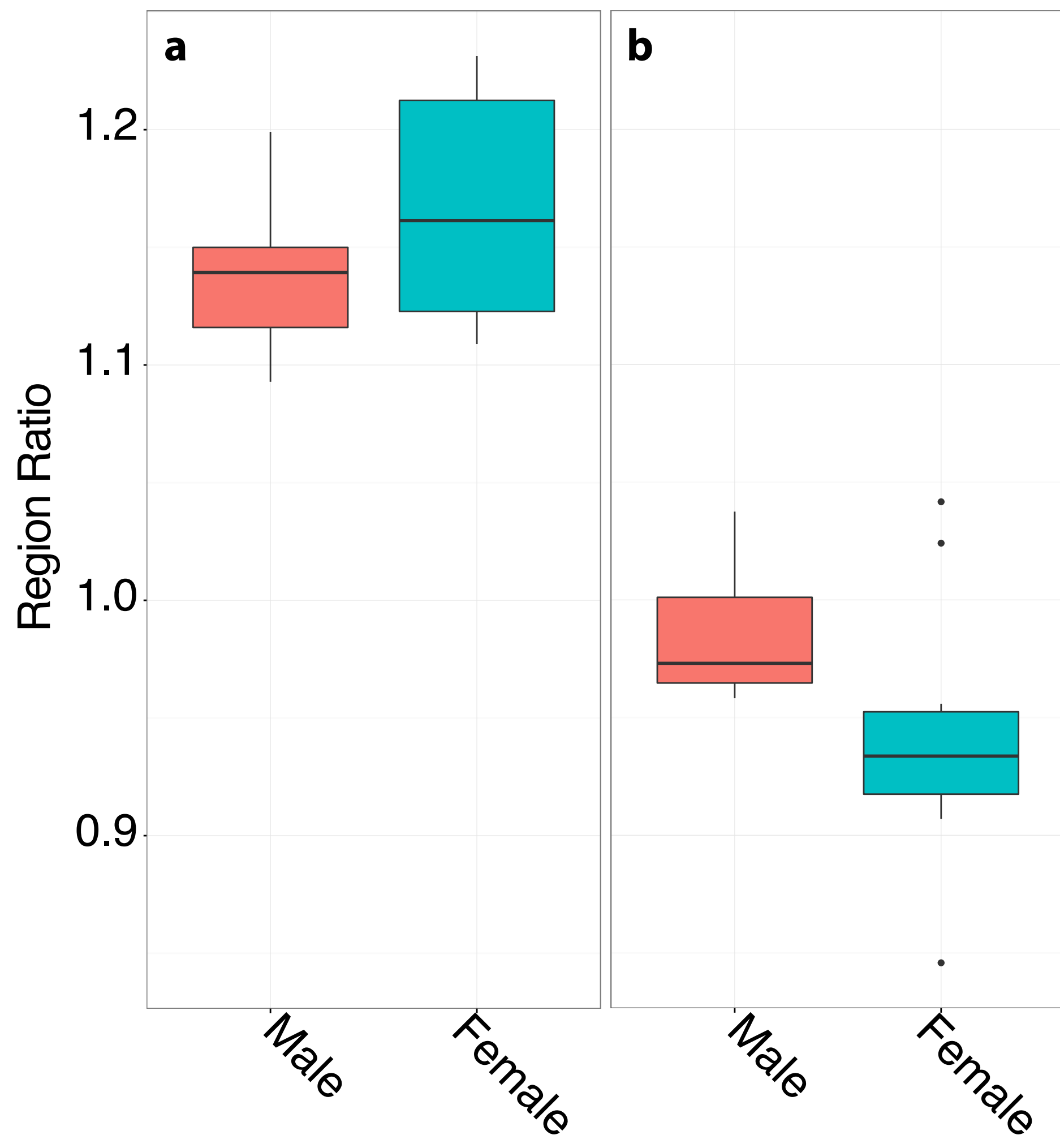

UNM Post-treatment

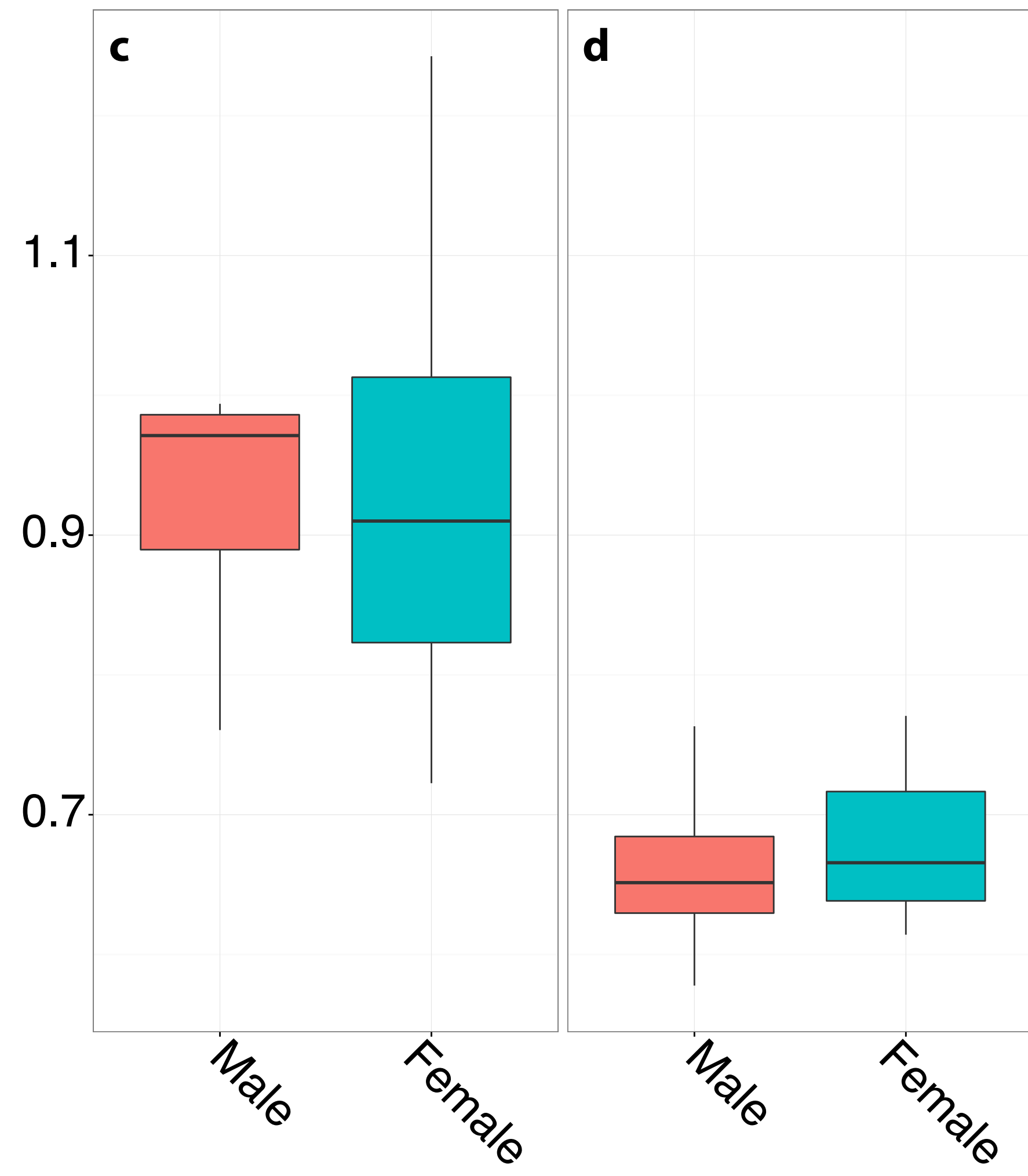

Merged Post-treatment

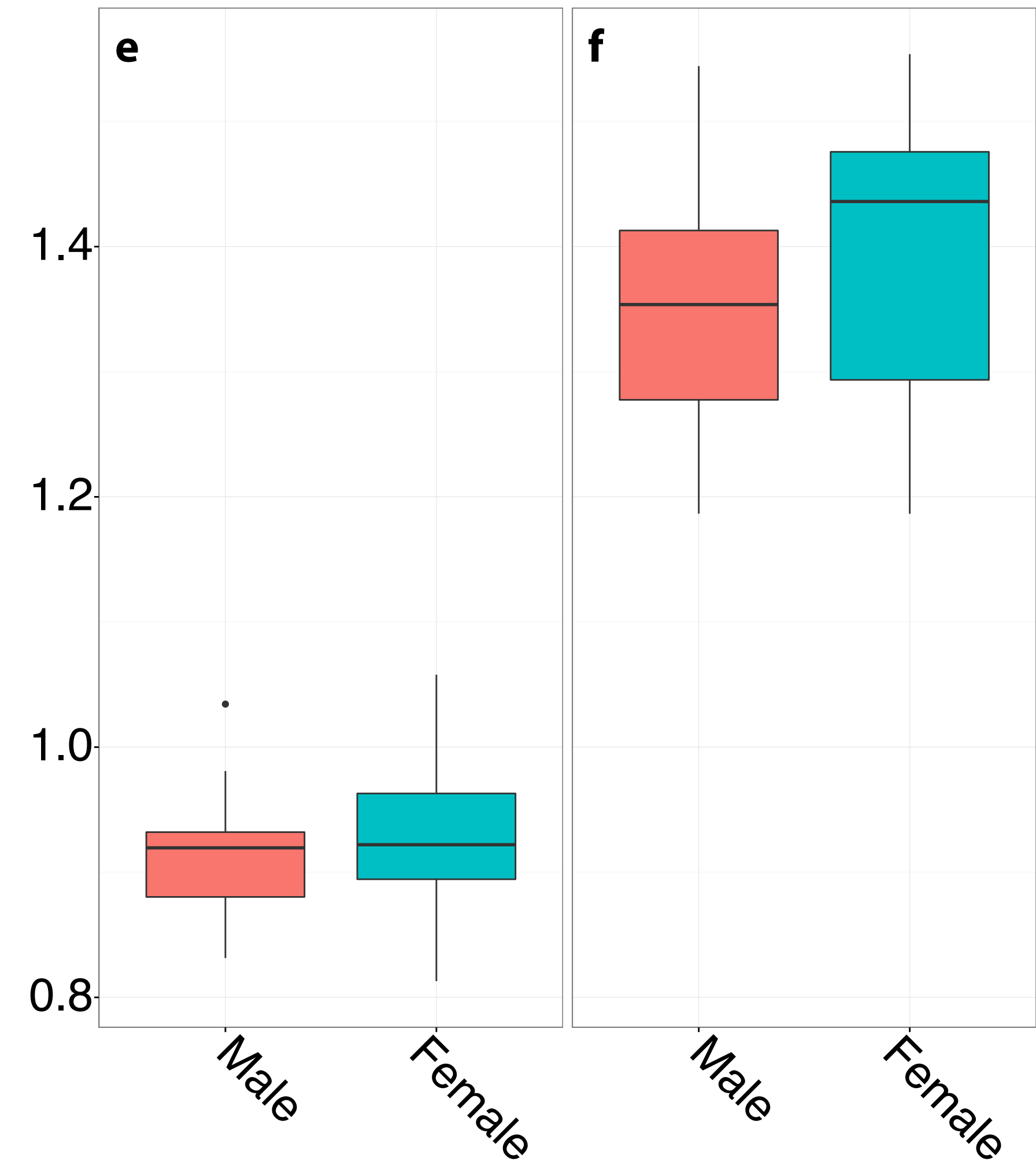

Supplement: Supplementary file 5 — Supplemental figure 4 [file 41398_2017_20_MOESM5_ESM.pdf]
